# ﻿Determining the best morphological characters for taxonomic identification of *Inga* species in the Colombian Andes using correlation, entropy and a discriminant index

**DOI:** 10.3897/phytokeys.267.172490

**Published:** 2025-12-12

**Authors:** Kelly T. Bocanegra-González, Thomas S. Prieto-González, Martin Pullan, Kyle G. Dexter, Hannah Atkins, R. Toby Pennington

**Affiliations:** 1 Research Group on Biodiversity and Dynamics of Tropical Ecosystems, University of Tolima, Ibagué, Colombia; 2 Royal Botanic Garden Edinburgh, Edinburgh, UK; 3 School of GeoSciences, University of Edinburgh, Edinburgh, UK; 4 Department of Life Sciences and Systems Biology, University of Turin, Turin, Italy; 5 Department of Geography, University of Exeter, Exeter, UK; 6 Department of Biology, Washington University in St Louis, St Louis, USA

**Keywords:** Character prioritisation, Colombian Andes, *
Inga
*, interactive key, taxonomic informatics

## Abstract

The genus *Inga* (Leguminosae, Caesalpinioideae) is a key component of tropical American forests, yet its high species diversity and morphological complexity hinder accurate identification, especially in montane environments. In this study, we evaluated 49 morphological characters (32 categorical and 17 continuous) from 73 *Inga* species occurring above 1000 m in Colombia. We applied a multi-criteria framework combining Spearman correlation, Shannon entropy and a discriminant index to prioritise diagnostic characters, based on redundancy, variability and discriminatory power. The results indicate that categorical characters, particularly stipule shape, indumentum type on both lamina surfaces and the petiole, interfoliar nectaries shape, leaflet apex and base shapes are the most informative for species identification and therefore received the highest scores. In contrast, continuous characters showed limited variation among species and were less useful and received lower scores. A usability test demonstrated higher accuracy and faster identifications when using the highest-scoring characters. This framework not only improves identification efficiency in field and herbarium settings but also offers a scalable model for intelligent, user-friendly taxonomic tools. As a complementary output, we present an open-access, interactive identification key, designed to improve the quality and reliability of *Inga* identifications across the Colombian Andes.

## ﻿Introduction

The genus *Inga* Mill. (Leguminosae, Caesalpinioideae) is a key component of tropical American forests, characterised by its high diversity (ca. 280 accepted species) and ecological significance (Pennington 1997; Endara and Jaramillo 2011; Palow et al. 2012; Bocanegra‐González et al. 2025). It is widespread across a range of tropical moist ecosystems. Most species occur below 1800 m above sea level, although some have been recorded at elevations exceeding 3000 m. These high-elevation species are particularly poorly explored, with limited information on their identity and distribution.

In recent years, important progress has been made in Colombia studying the genus (Romero and Alba-López 2005; Bocanegra-González et al. 2024, 2025). However, research on *Inga* faces several challenges, particularly in the high Andes (over 1000 m). These challenges arise from the poor knowledge of morphological variation amongst species and the absence of specific diagnostic characters that simplify identification and standardise identification keys.

According to Bisby (1970), a character is a concept shaped by criteria such as: *i*) morphological relevance, which ensures a link to plant structure; *ii*) variability, which highlights significant differences amongst species; *iii*) stability over ontogeny and under varying environmental conditions; *iv*) functionality, which reflects the plant’s adaptation and survival through the character; *v*) homology over homoplasy, which prioritises characters related to evolutionary relationships; *vi*) simplicity, which favours fewer, but more relevant characters in identification; and *vii*) accessibility, which ensures characters are observable and measurable.

For identifying *Inga*, most authors have used sets or subsets of ~ 74 morphological characters, both continuous and categorical (Leon 1966; Pennington 1997; Romero 2005; Romero and Alba-López 2005; Dexter et al. 2010; Genero et al. 2017; Soto et al. 2021; Bocanegra-González et al. 2024, 2025). These include the number of leaflet pairs, size of leaflets, presence and type of indumentum on both adaxial and abaxial leaf surfaces, wing presence and shape, nectary shape and diameter, inflorescence type, position and fruit type, amongst others (Suppl. material 1). Authors have often combined these characters differently (Leon 1966; Pennington 1997; Romero 2005; Romero and Alba-López 2005; Dexter et al. 2010; Genero et al. 2017; Soto et al. 2021; Bocanegra-González et al. 2024, 2025) and there is no general consensus on which characters to use for identifying species, at least in most cases. It is unlikely that a non-specialist would be able to manage the entire set of characters. In addition, some of these features are plastic and show high levels of variation within and amongst *Inga* species, geographical range, making field and herbarium identification challenging (Bocanegra‐González et al. 2025), contributing to a high percentage of misidentification in the genus (Dexter et al. 2010; Baker et al. 2017). Moreover, as for many tropical hyperdiverse groups, there is a lack of identification tools and traditional dichotomous keys are often not readily available to users (Särkinen et al. 2025).

Determining the most useful characters for identification can improve the efficiency and accuracy of identification in the field, in herbaria and when constructing identification keys (Bisby 1970; Talent et al. 2014; Rakouth et al. 2025). It can also enhance the performance of numerical procedures, such as artificial intelligence, for species identification (Mäder et al. 2021; Hart et al. 2023).

To reduce the dimensionality of highly multivariate, morphological data and improve numerical identification procedures, characters are often ranked, weighted or grouped (Talent et al. 2014). Importantly, the efficiency gained by prioritising characters may vary depending on the type of identification key being used. In dichotomous keys, where the path is fixed by the author, careful selection and ordering of characters is critical, as early mistakes can mislead the user in the identification process (Hagedorn et al. 2010). In contrast, multi-access keys (also known as polyclaves) allow users to choose the characters and may tolerate user scoring errors better and also allow identification when certain characters are unavailable (i.e. flowers and fruits) (Ohkawa 2000). Thus, character prioritisation has distinct implications depending on the key structure.

Approaches for character selection and species identification consider characters either collectively or in pairs, facilitating the construction of such keys (Talent et al. 2014; Rakouth et al. 2025). Some numerical methods involve frequency of divisions, entropy reduction, balanced characters, conflict reduction, error minimisation, time efficiency and weighted combinations. These approaches are based on set theory, Shannon entropy, proportional optimisation, discrimination analysis, probability, decision theory and multi-criteria optimisation (Lubischew 1962; Eades 1965; Hall 1967; Bisby 1970; Izenman 2008; Talent et al. 2014; Łysko et al. 2022). Despite the wide range of available methods, none has been universally or consistently adopted as a standardised approach. In addition, methods are rarely used together or compared.

Some of the commonly used numerical methods for character prioritisation reported in literature are Pearson’s correlations, aiming to reduce redundancy amongst character sets, that is, the overlap in characters that can complicate the identification process. However, it is not the best approach in numerical taxonomy and ecology, primarily because it measures linear relationships, while biological variation is often non-linear and multidimensional (Eades 1965; Hall 1967). Additionally, Pearson correlation analysis is sensitive to outliers, assumes normality in the data and loses information in categorical characters (Schober et al. 2018). Another correlation used is the Spearman’s, which measures the strength and direction of a monotonic relationship between two variables without assuming a specific frequency distribution. Unlike Pearson’s correlation, it does not require a linear relationship or interval-scale measurements and works for ordinal data (Hauke and Kossowski 2011).

Shannon entropy has been common in character selection (Eades 1965; Hall 1967; Bisby 1970; Talent et al. 2014) and can be understood as measuring the variability of elements within a given set, quantifying the number of yes/no questions needed to identify an element in a set (Carcassi et al. 2021). This method preserves more information about the distribution of data by avoiding the simplification that results from averaging values. Shannon entropy can be applied to non-linear systems, but it provides only a partial view of their complexity. For this reason, it is often recommended to use entropy in combination with other statistics, which can capture additional aspects of non-linear biological dynamics (Eskov et al. 2017).

Another common approach is linear discriminant analysis (LDA), which was developed to solve taxonomic (i.e. classification) issues using multiple botanical measurements (Brown and Wicker 2000; Izenman 2008). LDA assesses which characters identify unique species and has been used in multiple studies and is presently a popular method for training machine-learning (Lubischew 1962; Łysko et al. 2022). However, LDA assumes normality and requires multiple observations per class to estimate within-class variance, which limits its application when only a single observation per species is available or when data are only available as ranges of a continuous character per species. LDA can be replaced with a univariate discrimination index. This metric evaluates, for each character, the proportion of species that can be uniquely identified, based on that character alone, thus capturing its stand-alone discriminatory power. While it does not model multivariate separation as LDA does, it is robust for datasets with limited replicates, avoids violations of normality assumptions and still provides a quantitative basis for ranking characters in terms of their identification value.

In this study, we analysed a set of 49 continuous and categorical characters across Colombian Andean *Inga* species to identify those that are most effective for species identification. We employ a combined approach that includes Spearman’s correlation, Shannon entropy and a univariate discrimination index.

Our goal is to determine the most relevant set of characters required for identification, thereby enhancing the efficiency and accuracy of *Inga* identification. Finally, we present an interactive key that can be used with all the available characters, either including all characters or focusing on those prioritised as most informative.

## ﻿Materials and methods

### ﻿Data collection

**Figure 1.**
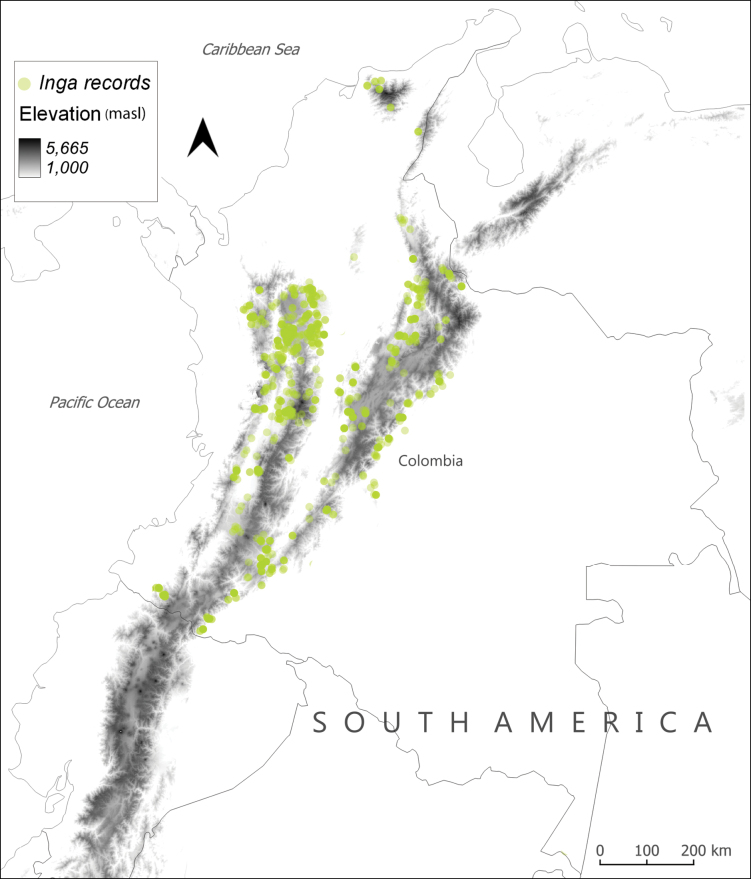
Curated *Inga* collections from the Colombian Andes obtained from GBIF range from elevations above 1000 m to a maximum of 3210 m a.s.l.

*Inga* species occurrences in Colombia above 1000 m were extracted from the Global Biodiversity Information Facility (GBIF.org 2024) (Fig. 1). These records were used to compile the list of species reported in the region. A total of 1339 records representing 73 species were included; 49 morphological characters for *Inga* (32 categorical and 17 continuous), selected from the 74 characters described in *Inga* studies were used in the analyses (Suppl. material 1). The 49 characters were chosen because they are consistently reported in literature (Leon 1966; Pennington 1997; Romero 2005; Romero and Alba-López 2005; Dexter et al. 2010; Genero et al. 2017; Soto et al. 2021; Bocanegra-González et al. 2024, 2025) and complemented with measurements taken from herbarium collections (COL, E, UDBC, TOLI), where all 73 species were represented and identifications confirmed.

In the case of categorical characters, their definitions were standardised across sources to ensure consistency (Suppl. material 2) and using as a base the plant glossary by Beentje (2010). We also considered the historical usage of terminology for these characters in the genus, especially that established by Pennington (1997). All the information was organised in a spreadsheet for subsequent analyses.

### ﻿Data analyses

The analyses were conducted in Google Colab, an interactive Python environment. All categorical variables were transformed into numerical values using an average relative frequency encoding method (Butcher and Smith 2020). For each character, the relative frequency of each state was computed by dividing the number of times that state appears by the total number of non-missing entries for that character. The value assigned to a species corresponds to the frequency of the state it exhibits, meaning that species sharing a common state receive the same numerical value. This approach reflects how common or rare each state is within the dataset. For multistate categorical characters, we calculated the relative frequency of each state across the dataset and used their average as the final encoded value. This approach preserved the information content of composite character states, while avoiding artificial inflation of dimensionality.

In the case of continuous characters, the metrics corresponded to the minimum and maximum values of each state. These were unified to establish ranges and were subsequently discretised into categorical classes using defined intervals, based on their observed ranges and biological relevance. Each continuous value was assigned to a bin. This binning approach facilitated integration with entropy and discrimination metrics. Subsequently, all character columns were normalised to a 0–1 range using Min–Max scaling to ensure comparability.

Pairwise character–character correlations were then computed with Spearman’s rank correlation using the “*spearmanr*” function from the “*scipy.stats*” module.

Shannon entropy was calculated for each variable to quantify the distribution of its character states. Higher entropy values indicate greater variability across states and, therefore, more potential for discrimination, whereas lower values reflect conserved characters that separate fewer species. Entropy was computed using the function “*scipy.stats.entropy*”, applied to the frequency distribution of states for each character.

Discrimination analysis was conducted using a custom univariate method. For each character, we calculated the proportion of species that could be uniquely identified, based on that character alone. This was done by grouping the dataset by character states and counting how many of those states were restricted to a single species using the “*pandas*” library. The number of exclusive states was then expressed as a percentage of the total number of species, defining the discrimination index as a measure of the standalone discriminative power of each character. A univariate approach was necessary to assess the relative contribution of each character in isolation, since the dataset is structured at the species level, with single composite records per species rather than multiple individuals.

Subsequently, a multiple weighting analysis was performed using three metrics: Spearman correlation, entropy and the discrimination index. These metrics were normalised using the Min-Max scaling method to ensure comparability. Additionally, a bilateral penalty was applied to the correlation metric to discourage both highly redundant and overly uncorrelated characters. Correlations below 0.10 and above 0.60 were penalised linearly, favouring characters with intermediate correlation values that contribute complementary, but non-redundant information to the dataset. Spearman correlation was weighted at 20% to account for redundancy among characters and to prevent highly correlated traits from dominating the analysis. Entropy received the highest weight (60%) because it reflects the overall variability and distribution of states across species, making it the most reliable indicator of consistent taxonomic signal. Finally, the discrimination index was weighted at 20% to incorporate the contribution of characters with uniquely identifying states, while avoiding overemphasis on characters informative only in a few species. Finally, an interactive key was built using Padme (Royal Botanic Garden Edinburgh) with the full character set.

### ﻿Usability assessment of the interactive key

To assess the practical utility of the interactive multi-access key developed in this study, we conducted a usability experiment comparing species identification performance when using the key with the full character set without prioritisation (unscored list) versus the prioritised characters (scored list). Ten participants with general botanical training, but no prior experience with *Inga* were selected. Each was provided with a set of three specimen photos reflecting observable morphological characters. Participants were randomly assigned to two groups (n = 5 and n = 5). Group A used the unscored list and Group B used the scored one (prioritised order). Each participant identified the same specimens using the same set of 49 characters.

Performance was assessed by comparing the mean identification time between the two-character lists and the number of characters used to reach the identification in each case. Both were evaluated using the Wilcoxon signed-rank test, a non-parametric method suitable for matched samples.

## ﻿Results

### ﻿Correlation

**Figure 2.**
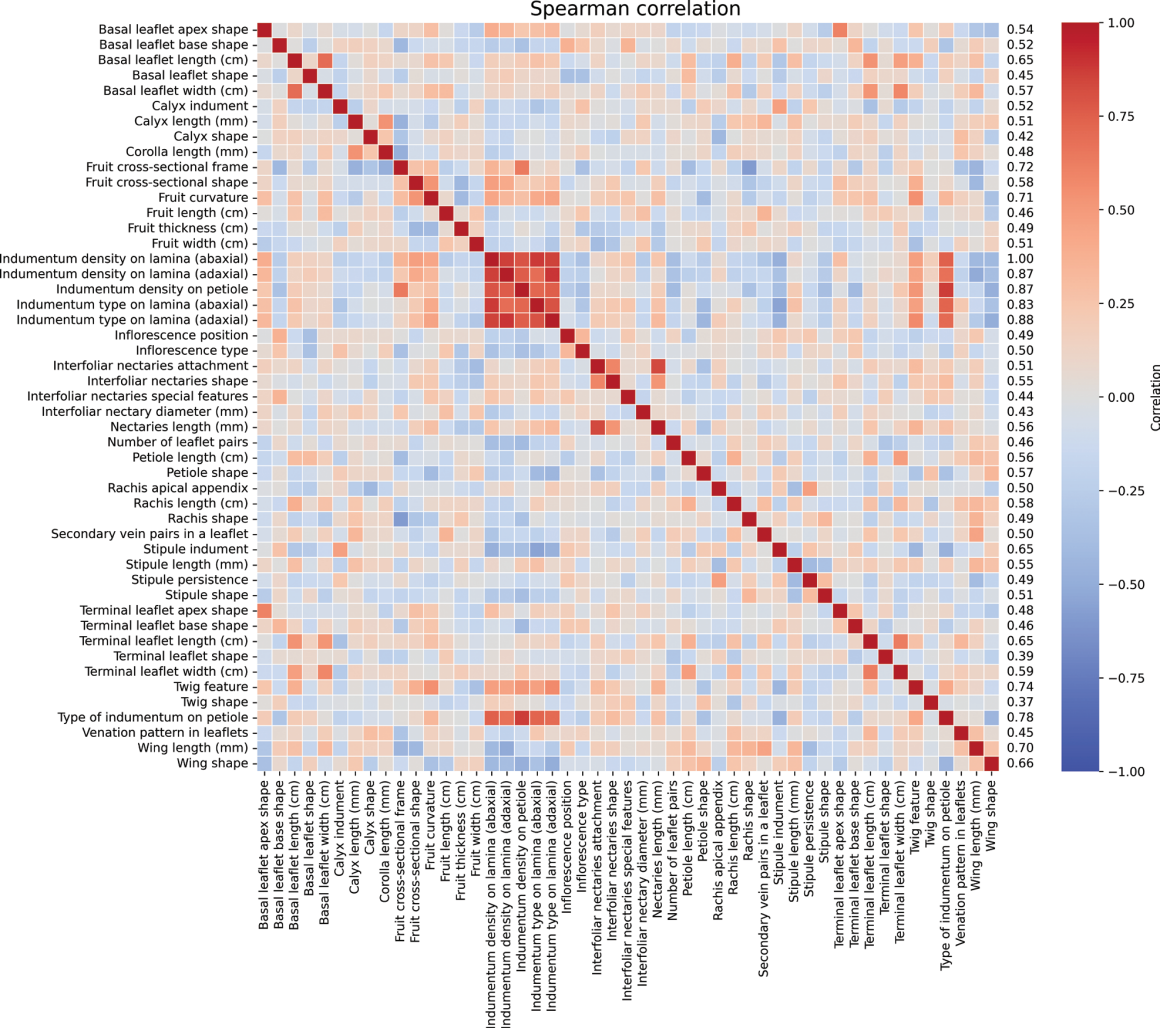
Spearman correlation matrix amongst 49 morphological characters of Colombian Andean *Inga* species. The heatmap shows the strength and direction of correlations between pairs of characters, with red indicating positive correlations and blue indicating negative ones. The values at the end of the axis represent the sum of the absolute correlation coefficients for each character, indicating their overall degree of association with other characters.

The correlations reflect the consistency and biological interdependence of the morphological characters studied, which is logical given the common origin and related functions of the plant structures that showed high correlation (> 60%) (Fig. 2). The consistent relationships between leaflet length and width for terminal and basal leaflets indicate proportional growth patterns. Additionally, correlations between indumentum type and density across abaxial and adaxial lamina surfaces suggest coordinated pubescence (Fig. 2). Similarly, correlation between calyx and corolla length, as well as fruit length, width and thickness, reflect coordinated variation in these characters (Fig. 2). Beyond these groups, most characters showed low to moderate associations, indicating that they vary largely independently and thus provide complementary information for species delimitation in *Inga*.

### ﻿Shannon entropy

**Figure 3.**
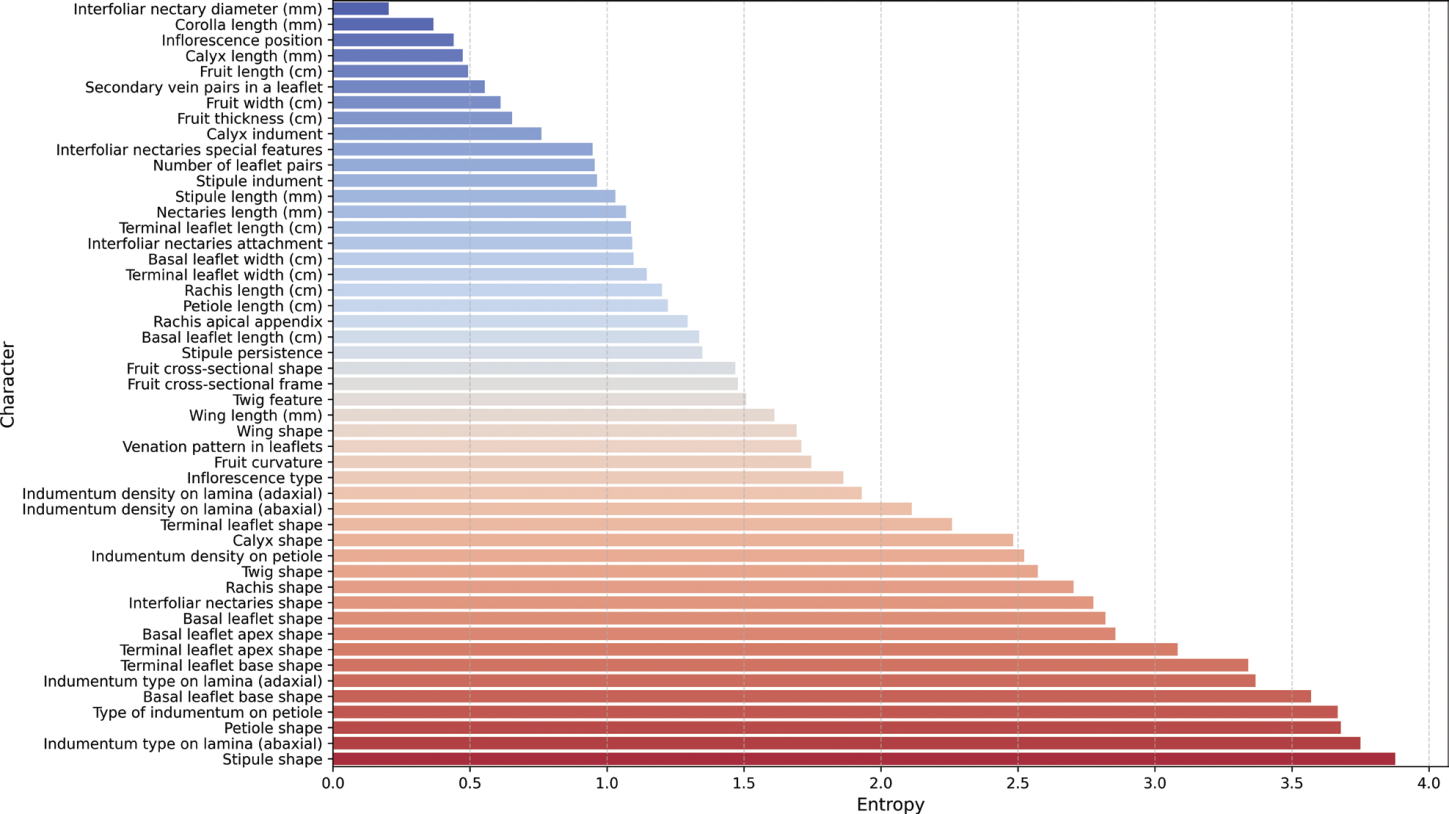
Shannon entropy values for 49 morphological characters of Colombian Andean *Inga* species. The barplot shows the relative information content of each character, with higher entropy values indicating greater variability and discriminatory power across species.

The characters showing the highest entropy values (above 3) are stipule shape, indumentum type on lamina (abaxial), type of indument in petiole, petiole shape and basal leaflet base shape (Fig. 3). This high entropy amongst structural descriptors reflects a wide diversity in how these forms are distributed across *Inga*. In contrast, characters like interfoliar nectary diameter, corolla length, calyx length, inflorescence position and fruit length exhibit lower entropy (below 0.5) (Fig. 3). The lowest entropy is related to characters that are highly conserved across the sampled taxa, which means they do not separate as many species by themselves. It is worth noting that metric characters were transformed into discrete categories in the dataset, which may reduce their range of variation and thus lower their entropy values. Overall, the results reveal a broad spectrum of entropy values from nearly invariant characters to highly variable ones underscoring that only a subset of descriptors contributes strongly to species delimitation. In this dataset, characters with entropy values above 2 clearly form the uppermost group in the distribution and were, therefore, considered the most informative.

### ﻿Discrimination analysis

Shape-related characters, such as stipule shape (19.44%), basal leaflet base shape (18.06%), indumentum type on lamina (abaxial) (13.89%), interfoliar nectaries shape and type of indumentum on petiole (12.50%), exhibited the highest discriminant index values (> 12%), highlighting their strong contribution to species delimitation (Table 1). These characters are categorical descriptors that tend to remain stable within species, but differ clearly amongst taxa, making them especially informative for taxonomic identification. A second group of characters showed intermediate contributions (4–11%), including basal leaflet apex shape, terminal leaflet apex shape, terminal leaflet base shape and several additional shape-related features across different structures (Table 1). Although less powerful individually, they still provide relevant discriminatory information. In contrast, most metric characters (e.g. interfoliar nectary diameter (mm), fruit length (cm), corolla length (mm), nectaries length (mm)) consistently scored very low (≈ 1.39% or null) (Table 1), suggesting that continuous measurements, once grouped into size intervals, capture less interspecific variation and, therefore, contribute little to species separation in this dataset. Overall, the discrimination analysis highlights that a limited set of categorical structural characters accounts for most of the univariate discriminative power in *Inga*, supporting species delimitation.

**Table 1.** Discriminant analysis of 49 morphological characters in Colombian Andean *Inga* species. The table shows the contribution of each character to species discrimination, measured as the Discriminant Index (%). Higher values indicate a stronger influence on the separation amongst species in univariate space.

Character	Discrimination Index %	Character	Discrimination Index %
Stipule shape	19.44	Fruit length (cm)	1.39
Basal leaflet base shape	18.06	Terminal leaflet length (cm)	1.39
Indumentum type on lamina (abaxial)	13.89	Stipule length (mm)	1.39
Interfoliar nectaries shape	12.50	Corolla length (mm)	1.39
Type of indumentum on petiole	12.50	Stipule persistence	0.00
Basal leaflet apex shape	11.11	Interfoliar nectaries attachment	0.00
Terminal leaflet apex shape	9.72	Rachis apical appendix	0.00
Terminal leaflet base shape	8.33	Stipule indument	0.00
Petiole shape	8.33	Nectaries length (mm)	0.00
Rachis shape	6.94	Fruit cross-sectional frame	0.00
Indumentum type on lamina (adaxial)	6.94	Calyx indument	0.00
Inflorescence position	5.56	Indumentum density on lamina (adaxial)	0.00
Inflorescence type	5.56	Indumentum density on lamina (abaxial)	0.00
Terminal leaflet shape	5.56	Fruit cross-sectional shape	0.00
Indumentum density on petiole	5.56	Wing length (mm)	0.00
Twig shape	4.17	Petiole length (cm)	0.00
Basal leaflet shape	2.78	Terminal leaflet width (cm)	0.00
Venation pattern in leaflets	2.78	Number of leaflet pairs	0.00
Fruit thickness (cm)	2.78	Rachis length (cm)	0.00
Fruit curvature	1.39	Basal leaflet length (cm)	0.00
Calyx shape	1.39	Calyx length (mm)	0.00
Interfoliar nectary diameter (mm)	1.39	Secondary vein pairs in a leaflet	0.00
Twig feature	1.39	Basal leaflet width (cm)	0.00
Wing shape	1.39	Fruit width (cm)	0.00
Interfoliar nectaries special features	1.39		

### ﻿Multiple weighting analysis

The weighted scores indicate that categorical characters concentrate the highest values (Table 2). Stipule shape (1), basal leaflet base shape (0.94), type of indumentum on petiole (0.88) and indumentum type on lamina (abaxial) (0.86) occupy the top ranks, followed closely by petiole shape, terminal leaflet base and apex shape, basal leaflet apex shape and interfoliar nectaries shape (0.74–0.85). These characters emerge as the strongest sources of taxonomic signal once correlation, entropy and discrimination are jointly considered. In contrast, metric characters related to organ dimensions, such as corolla length, leaflet length and width and interfoliar nectary diameter, amongst others, consistently fall to the bottom (< 0.40), confirming their limited contribution under this integrative framework. Between these extremes, there is a group of characters with intermediate scores (0.40–0.73).

**Table 2.** Weighted scores for 49 morphological characters of Colombian Andean *Inga* species, based on a multi-criteria analysis. The score reflects the overall contribution of each character to species identification, integrating four metrics: correlation, entropy and discrimination index. Higher scores indicate characters that are more informative and consistent across species. Characters are ranked from most to least informative.

Character	Weighted Score	Character	Weighted Score
Stipule shape	1.00	Indumentum density on lamina (adaxial)	0.40
Basal leaflet base shape	0.94	Basal leaflet length (cm)	0.39
Type of indumentum on petiole	0.88	Rachis apical appendix	0.38
Indumentum type on lamina (abaxial)	0.86	Rachis length (cm)	0.37
Petiole shape	0.85	Petiole length (cm)	0.37
Terminal leaflet base shape	0.80	Terminal leaflet length (cm)	0.37
Terminal leaflet apex shape	0.75	Terminal leaflet width (cm)	0.36
Basal leaflet apex shape	0.74	Stipule length (mm)	0.36
Interfoliar nectaries shape	0.74	Basal leaflet width (cm)	0.35
Rachis shape	0.72	Interfoliar nectaries attachment	0.35
Indumentum type on lamina (adaxial)	0.68	Nectaries length (mm)	0.35
Basal leaflet shape	0.67	Interfoliar nectaries special features	0.35
Calyx shape	0.58	Number of leaflet pairs	0.34
Terminal leaflet shape	0.54	Stipule indument	0.33
Inflorescence type	0.54	Indumentum density on lamina (abaxial)	0.32
Twig shape	0.53	Fruit thickness (cm)	0.31
Indumentum density on petiole	0.51	Inflorescence position	0.31
Venation pattern in leaflets	0.48	Calyx indument	0.30
Fruit curvature	0.47	Fruit width (cm)	0.28
Wing shape	0.47	Fruit length (cm)	0.27
Wing length (mm)	0.44	Secondary vein pairs in a leaflet	0.26
Twig feature	0.44	Corolla length (mm)	0.25
Fruit cross-sectional frame	0.42	Calyx length (mm)	0.25
Fruit cross-sectional shape	0.41	Interfoliar nectary diameter (mm)	0.20
Stipule persistence	0.40		

### ﻿Identification characters’ guide for *Inga* species, based on weighted scores

The identification of an *Inga* species, according to our analysis, should begin with the highest-value characters, based on the weighted scores (Table 2) and progressively move to intermediate and lower-ranked ones. Prioritising characters in this order can efficiently separate the species. Subsequently, less discriminatory characters (those with intermediate weighted scores) should be considered. At the same time, characters with high weighted scores, based on metrics, cannot be directly used unless their biological relevance is interpreted within a specific context. For instance, measuring nectary length (mm) would be irrelevant if the species in question has the more common short cup-shaped nectary. This character would work very well for tubular nectaries.

The multi-access interactive key developed [https://padme.rbge.org.uk/keys/welcome/inga] can be used by selecting any input character; however, the identification process becomes more efficient when using the highest-scored characters. The comparison of mean identification times between the scored and unscored character lists showed no statistically significant difference (Wilcoxon signed-rank test, p = 0.50). Nonetheless, participants using the scored list (Group B) tended to complete the identifications more quickly (6.3 min vs. 12.5 min in Group A) and with fewer characters (4.5 vs. 8.3; Wilcoxon signed-rank test, p = 0.25). Taken together, these results suggest that prioritising characters can reduce both the effort and time needed for species identification, even if the differences were not statistically significant.

## ﻿Discussion

This study employed Spearman correlation, Shannon entropy and a discriminant analysis to identify the most effective characters for species identification in the genus *Inga* from the Colombian Andes (above 1000 m), using 49 morphological characters (32 categorical and 17 continuous). This study demonstrates the importance of combining multiple analytical approaches to optimise the selection of diagnostic characters. This is important because no single method is sufficiently accurate on its own in plant identification. This approach reflects a broader trend in modern taxonomy and biodiversity informatics, where no single metric fully captures the complexity of morphological variation. Combining complementary methods, as seen in recent deep learning models for species recognition, improves overall robustness (Carranza-Rojas et al. 2017; Särkinen et al. 2025).

The selected approach highlights the biological consistency (correlation) of certain characters that are closely related in their structural development. It not only optimises identification, but also reduces the time and effort required for data collection in the field and herbaria. For example, in the case of *Inga*, recording the indument type on both lamina surfaces (adaxial and abaxial) is unnecessary, as is recording the separate lengths and widths of the leaflets. While character measurement remains essential for the recognition of *Inga* species, this approach focuses on identifying the most efficient combination of characters to be measured and recorded, avoiding unnecessary redundancy and improving the overall diagnostic process.

Additionally, the Shannon entropy results highlight those categorical characters that are the primary contributors to distinguishing species within the dataset. In contrast, continuous characters show lower entropy and, thus, less discriminative power, likely because their values were grouped into size intervals. Although different interval definitions could be tested, in this study, we applied a uniform binning scheme to maintain consistency across characters and to align with standard morphological ranges reported in literature.

The discriminant index indicates that a few categorical characters, such as stipule shape, basal leaflet base shape and terminal leaflet apex shape, provide most of the taxonomic resolution in *Inga*. This pattern reinforces that those qualitative structural characters are the most informative for species delimitation in the genus.

Taken together, the previous results provide clear guidance for how characters should be prioritised when constructing identification tools. Furthermore, the choice and order of characters have different implications depending on the type of key. In dichotomous keys, where the sequence is fixed, prioritising stable and easy-to-score characters at the beginning can help prevent misidentifications (Hagedorn et al. 2010). In contrast, multi-access keys allow users to bypass problematic or ambiguous characters, making them more tolerant to variation and observational difficulties (Ohkawa 2000; Särkinen et al. 2025). The methodology proposed here is, therefore, particularly valuable for improving the structure of dichotomous keys, while also enhancing the efficiency of user-driven identification tools. Ultimately, this approach is intended to facilitate accurate and accessible species identification in *Inga*.

The scoring generated here highlights that the vegetative characters are far more important than reproductive ones in the case of *Inga*, reinforcing the need to prioritise vegetative characters where possible. Their relevance is further supported by other studies, where they have proven extremely useful in the development of tropical dynamic keys (Särkinen et al. 2025).

Furthermore, reliance on statistical methods that assume data normality may introduce biases, especially in continuous datasets. Future studies would benefit from integrating these results into the development of automated tools, such as intelligent systems for species identification, based on prioritised characters, which could revolutionise the practical taxonomy of the genus (Wäldchen et al. 2018; Mäder et al. 2021; Hart et al. 2023).

In addition, we highlight the issue of how characters are treated prior to any statistical analysis. The standardisation of characters, no matter how carefully designed, remains partly subjective, as many have undergone changes in their definitions since the most relevant taxonomic treatments of *Inga*, for instance, the type of induments (Leon 1966; Pennington 1997). This highlights a persistent challenge: ensuring that, despite conceptual differences across studies, the historical terminology applied to characters can be unified and consistently applied. Such unification is essential to preserve continuity with past work, while enabling comparability and reliability in current and future identification tools. Here, we integrate these characters and refine state definitions, expanding them for species that appeared constrained by characters not well accommodated within the pre-established categories (Leon 1966; Pennington 1997; Romero 2005; Romero and Alba-López 2005; Dexter et al. 2010; Genero et al. 2017; Soto et al. 2021; Bocanegra-González et al. 2024, 2025).

The character prioritisation framework developed also provides a foundation for future integration with AI-based identification tools. Recent advances in computer vision and mobile applications have demonstrated the potential of intelligent systems to facilitate species recognition even for non-specialists (Carranza-Rojas et al. 2017; Wäldchen et al. 2018). Tools such as Flora Incognita (https://floraincognita.com/) already leverage similar principles to guide user input and could benefit from datasets structured around character consistency and discriminatory power. This positions our results not only as a taxonomic resource, but also as a bridge towards scalable, user-friendly biodiversity tools. This study presents a replicable model for prioritising taxonomic characters in diverse groups such as *Inga*. The interactive key constructed from these results has the potential to facilitate species identification in the field and herbariums, especially for non-specialist users.

## ﻿Additional information

### Use of AI

AI was used exclusively for language editing.

### Author ORCIDs

Kelly T. Bocanegra-González https://orcid.org/0000-0001-7177-5856

Thomas S. Prieto-González https://orcid.org/0009-0002-8657-607X

Martin Pullan https://orcid.org/0000-0002-4452-6026

Kyle G. Dexter https://orcid.org/0000-0001-9232-5221

Hannah Atkins https://orcid.org/0000-0002-3523-1883

R. Toby Pennington https://orcid.org/0000-0002-8196-288X

### Data availability

All of the data that support the findings of this study are available in the main text or Supplementary Information.

## Supplementary materials

Supplementary material 1 Morphological characters recorded for *Inga* Data type docx Explanation note 74 morphological characters recorded for *Inga*, including their possible states and indication of which were included in the analysis. https://binary.pensoft.net/file/1491285This dataset is made available under the Open Database License (http://opendatacommons.org/licenses/odbl/1.0/). The Open Database License (ODbL) is a license agreement intended to allow users to freely share, modify, and use this Dataset while maintaining this same freedom for others, provided that the original source and author(s) are credited.Kelly T. Bocanegra-González, Thomas S. Prieto-González, Martin Pullan, Kyle G. Dexter, Hannah Atkins, R. Toby Pennington

Supplementary material 2 Categorical characters were standardised following Beentje’s Plant Glossary (2010) and Pennington’s (1997) taxonomic treatment of *Inga* Data type docx Explanation note Illustrated glossary of categorical characters for *Inga*, showing standardised states (e.g., twig shapes, stipule forms, petiole and rachis types, indumentum categories, nectary forms, inflorescence types, calyx shapes, and fruit morphologies). https://binary.pensoft.net/file/1491286This dataset is made available under the Open Database License (http://opendatacommons.org/licenses/odbl/1.0/). The Open Database License (ODbL) is a license agreement intended to allow users to freely share, modify, and use this Dataset while maintaining this same freedom for others, provided that the original source and author(s) are credited.Kelly T. Bocanegra-González, Thomas S. Prieto-González, Martin Pullan, Kyle G. Dexter, Hannah Atkins, R. Toby Pennington
