# Supplementary material for: ﻿Determining the best morphological characters for taxonomic identification of Inga species in the Colombian Andes using correlation, entropy and a discriminant index
Source: PhytoKeys. 2025 Dec 12;267:345–60. doi: 10.3897/phytokeys.267.172490 (PMC12717514; doi:10.3897/phytokeys.267.172490)
Supplement: Supplementary material 1 — Morphological characters recorded for Inga [file phytokeys-267-345_article-172490__-s001.docx]

**Determining the best morphological characters for taxonomic identification of *Inga* species in the Colombian Andes using correlation, entropy, and a discriminant index**

**Supplementary Material 1**

**Table 1.** Morphological characters recorded for *Inga*

| n° | Character | States | Included |
| --- | --- | --- | --- |
| 1 | Basal leaflets apex shape | Acuminate, Acute, Attenuate, Cuspidate, Obtuse, Rounded, Mucronate | X |
| 2 | Basal leaflets base shape | Acute, Attenuate, Cordate, Cuneate, Obtuse, Rounded, Truncate | X |
| 3 | Basal leaflets length | Quantitative measurement in cm | X |
| 4 | Basal leaflets shape | Elliptic, Lanceolate, Oblanceolate, Obovate, Ovate, Rhomboid | X |
| 5 | Basal leaflets width | Quantitative measurement in cm | X |
| 6 | Bract length | Quantitative measurement in cm |  |
| 7 | Bract shape | Linear, Spatulate, Geniculate, Pileate, Lanceolate |  |
| 8 | Calyx bud length | Quantitative measurement in mm |  |
| 9 | Calyx bud lobes number | Calyx bud lobes number |  |
| 10 | Calyx bud shape | Open, Close |  |
| 11 | Calyx indument | Present, Absent | X |
| 12 | Calyx length | Quantitative measurement in mm | X |
| 13 | Calyx shape | Cup-shaped, Funnel-shaped, Lateral slit, Tubular | X |
| 14 | Carpel number | Carpel number |  |
| 15 | Corolla length | Quantitative measurement in mm | X |
| 16 | Diameter young shoots | Quantitative measurement in cm |  |
| 17 | Floral rachis indument | Present, Absent |  |
| 18 | Floral rachis length | Quantitative measurement in mm |  |
| 19 | Flower colour | White, Yellow-cream |  |
| 20 | Free filaments length | Quantitative measurement in mm |  |
| 21 | Fruit cross-sectional frame | Smooth, Channelled, Winged margins | X |
| 22 | Fruit cross-sectional shape | Flat, Cylindrical, Quadrangular | X |
| 23 | Fruit curvature | Straight, Convex, Spirally/Twisted | X |
| 24 | Fruit length | Quantitative measurement in cm | X |
| 25 | Fruit thickness | Quantitative measurement in cm | X |
| 26 | Fruit width | Quantitative measurement in cm | X |
| 27 | Inflorescence position | Axillary, Cauliflorous, Ramiflorous | X |
| 28 | Inflorescence type | Capitata, Raceme, Spike, Subcapitata, Umbellate | X |
| 29 | Interfoliar nectaries attachment | Sunken, Sessile, Stalked | X |
| 30 | Interfoliar nectaries diameter | Quantitative measurement in mm | X |
| 31 | Interfoliar nectaries length | Quantitative measurement in mm | X |
| 32 | Interfoliar nectaries shape | Cup-shaped, Patelliform, Pulvinate, Tubular with expanded head, Tubular, Transversely compressed (kidney-shape) | X |
| 33 | Interfoliar nectaries special features | Absent, Additional nectaries on the leaflets, Multiple nectaries in the internode, Thick-walled | X |
| 34 | Lamina (abaxial) indumentum | Crisped-pubescent, Glabrous, Hirsute, Hispid, Midrib puberulous, Midrib pubescent, Minute reddish glandular hairs, Puberulous, Pubescent, Setose, Stiffy, Strigose, Subglabrous, Tomentose | X |
| 35 | Lamina (abaxial) indumentum density | Dense, Moderately dense, Sparse | X |
| 36 | Lamina (adaxial) indumentum | Crisped-pubescent, Glabrous, Hirsute, Hispid, Midrib hispid, Midrib puberulous, Midrib pubescent, Midrib tomentose, Minute reddish glandular hairs, Pubescent, Setose, Stiffy, Strigose, Subglabrous, Velutinous, Villose | X |
| 37 | Lamina (adaxial) indumentum density | Dense, Moderately dense, Scattered, Sparse | X |
| 38 | Legume margins | Present, Absent |  |
| 39 | Legume venation | Present, Absent |  |
| 40 | Number of flowers per inflorescence | Number of flowers |  |
| 41 | Number of leaflet pairs | Number of leaflets pairs | X |
| 42 | Pedicel length | Quantitative measurement in mm |  |
| 43 | Peduncle indumentum | Absent, Present |  |
| 44 | Peduncle length | Quantitative measurement in mm |  |
| 45 | Petiole indument | Crisped-pubescent, Glabrous, Hispid, Puberulous, Pubescent, Setose, Stiffy, Strigillose, Subglabrous, Tomentose, Velutinous, Villose | X |
| 46 | Petiole indument density | Coarse, Dense, Moderately dense, Sparse | X |
| 47 | Petiole length | Quantitative measurement in cm | X |
| 48 | Petiole shape | Marginate, channelled, Semiterete, Terete, Winged | X |
| 49 | Petiolule length | Quantitative measurement in cm |  |
| 50 | Rachis apical appendix | Absent, Present | X |
| 51 | Rachis length | Quantitative measurement in cm | X |
| 52 | Rachis shape | Marginate, Channelled, Semiterete, Terete, Winged | X |
| 53 | Rachis wing length | Quantitative measurement in mm | X |
| 54 | Rachis wing shape | Winged along the entire length of the rachis, Winged only below the internode, Wings extending from below the internode to beyond the mid-rachis, Wings extending from below the internode to mid-rachis | X |
| 55 | Secondary vein pairs in a leaflet | Number of pairs | X |
| 56 | Seed length | Quantitative measurement in cm |  |
| 57 | Seed thickness | Quantitative measurement in cm |  |
| 58 | Seed width | Quantitative measurement in cm |  |
| 59 | Stamen exserted | Exserted, Equal to corolla |  |
| 60 | Stamens number | Stamens number |  |
| 61 | Staminal tube length | Quantitative measurement in mm |  |
| 62 | Stipule indument | Absent, Present | X |
| 63 | Stipule length | Quantitative measurement in mm | X |
| 64 | Stipule persistence | Caducous, Persistent |  |
| 65 | Stipule shape | Cordate, Elliptic, Falcate, Lanceolate, Linear, Oblanceolate, Oblong, Obovate, Ovate, Spatulate, Suborbicular, Subulate | X |
| 66 | Terminal leaflet apex shape | Acuminate, Acute, Attenuate, Cuspidate, Obtuse, Rounded, Mucronate | X |
| 67 | Terminal leaflet base shape | Acute, Attenuate, Cordate, Cuneate, Obtuse, Rounded, Truncate | X |
| 68 | Terminal leaflet length | Quantitative measurement in cm | X |
| 69 | Terminal leaflet shape | Elliptic, Lanceolate, Oblanceolate, Oblong, Obovate, Ovate | X |
| 70 | Terminal leaflet width | Quantitative measurement in cm | X |
| 71 | Twig feature | Indumentum, Lenticelated, Lenticelated and indument | X |
| 72 | Twig shape | Channelled, Quadrangular, Terete, With decurrent lines - Striate | X |
| 73 | Venation pattern in leaflets | Brochidodromous, Eucamptodromous, Eucamptodromous to brochidodromous | X |
| 74 | Young shoots | Indumentum, Lenticelated, Lenticelated and indument |  |
