# Supplementary material for: ﻿Determining the best morphological characters for taxonomic identification of Inga species in the Colombian Andes using correlation, entropy and a discriminant index
Source: PhytoKeys. 2025 Dec 12;267:345–60. doi: 10.3897/phytokeys.267.172490 (PMC12717514; doi:10.3897/phytokeys.267.172490)
Supplement: Supplementary material 2 — Categorical characters were standardised following Beentje’s Plant Glossary (2010) and Pennington’s (1997) taxonomic treatment of Inga [file phytokeys-267-345_article-172490__-s002.docx]

**Determining the best morphological characters for taxonomic identification of Inga species in the Colombian Andes using correlation, entropy, and a discriminant index**

**Supplementary Material 2**

Categorical characters were standardised following Beentje’s Plant Glossary (2010) and Pennington’s (1997) taxonomic treatment of *Inga*.

1. Twigs
   1. Shape


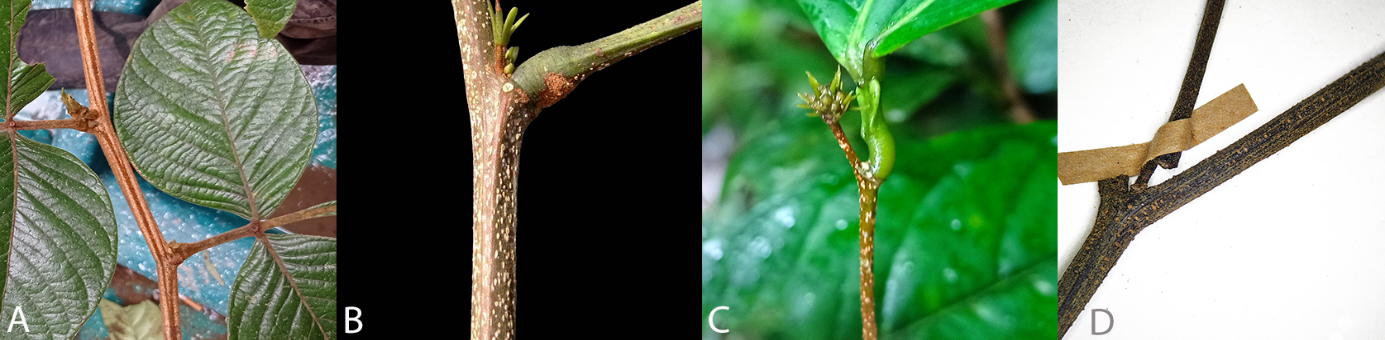


Figure 1. Twigs shapes through *Inga*. A. Channelled; B. Quadrangular; C. Terete; D. With recurrent lines-striate.

- 1. Features


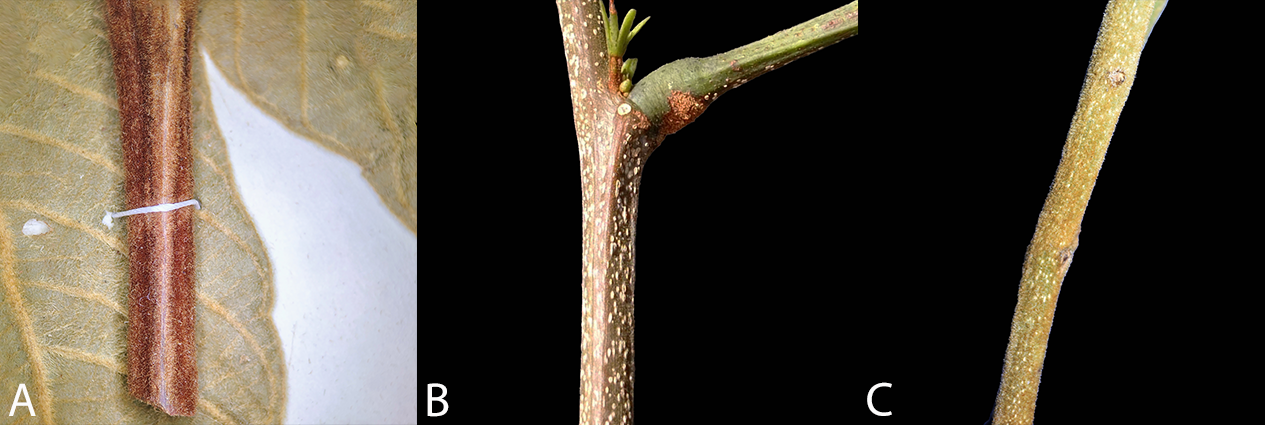


Figure 2. Twigs features through *Inga*. A. Indumentum; B. Lenticelate; C. Lenticelated and indument.

1. Stipules

2.1. Stipules shape


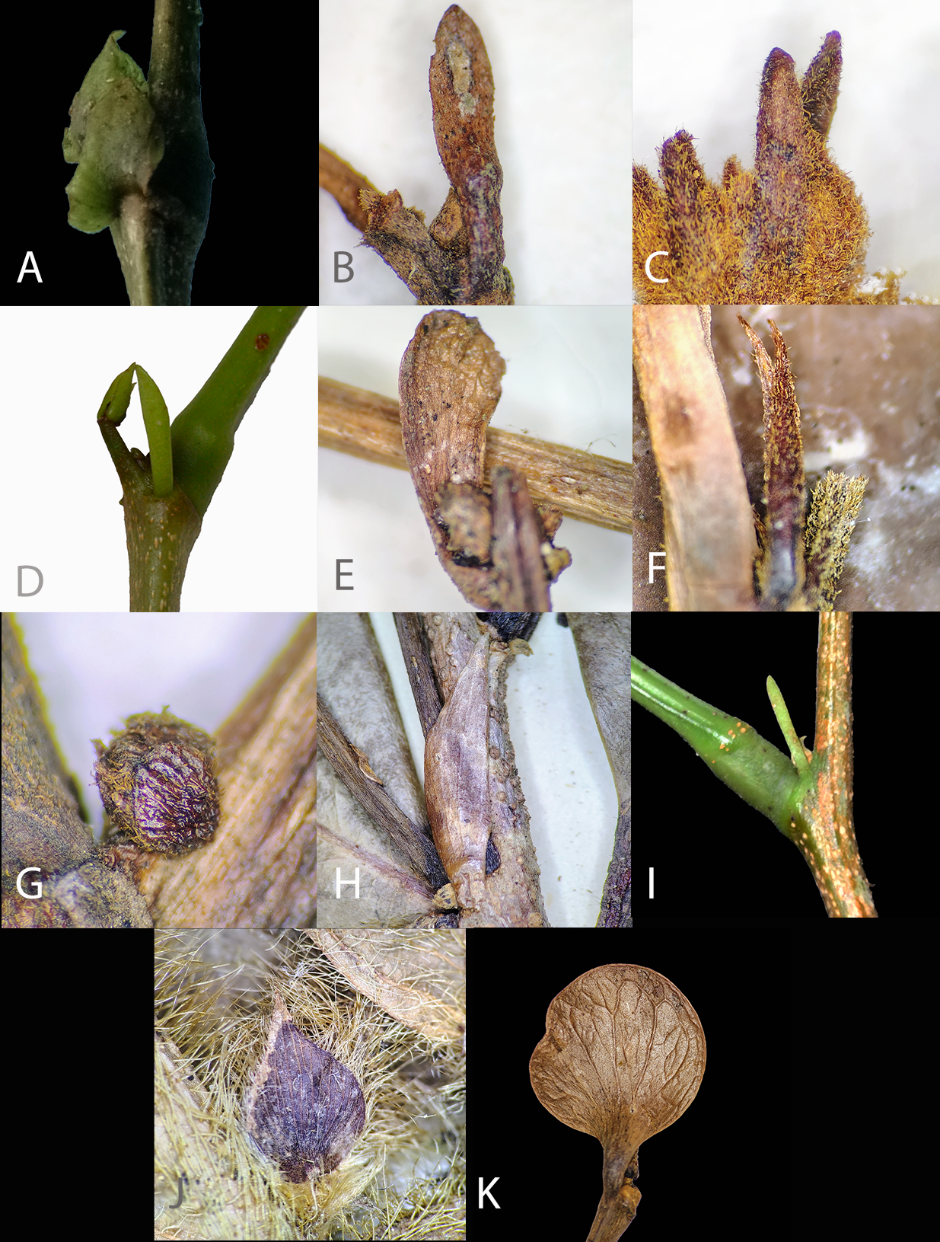


Figure 3. Stipules shapes through *Inga*. A. Cordate; B. Elliptic; C. Oblong/Oblanceolate; D. Lanceolate; E. Obovate; F. Subulate; G. Spatulate; H. Falcate; I. Linear; J. Ovate; K. Suborbicular.

1. Petiole
   1. Petiole shape


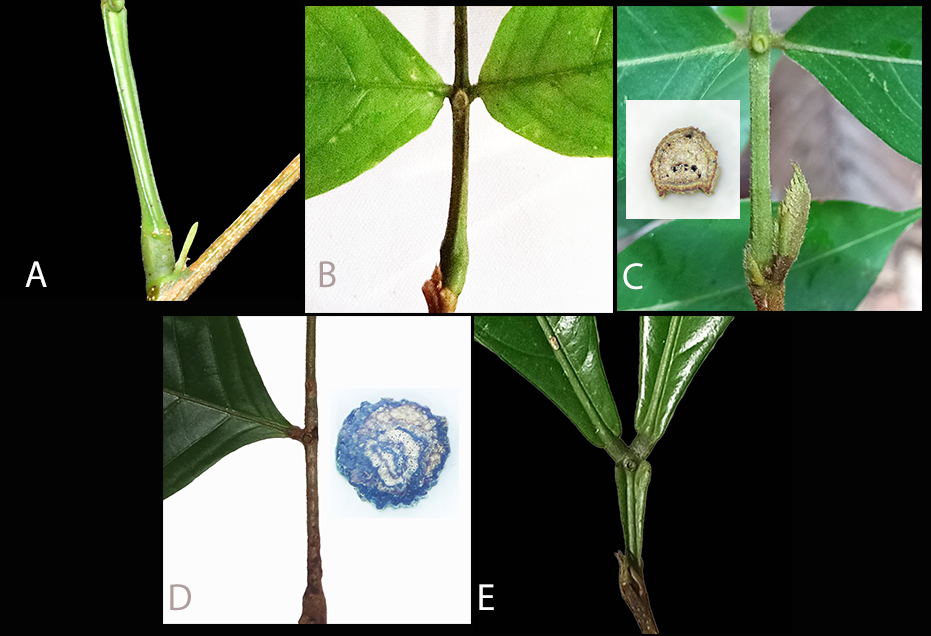


Figure 4. Petiole shapes through *Inga*. A. Channelled; B. Marginate; C. Semiterete (on the right transversal cross-section); D. Terete (on the left transversal cross-section); E. Winged.

1. Rachis
   1. Rachis shape


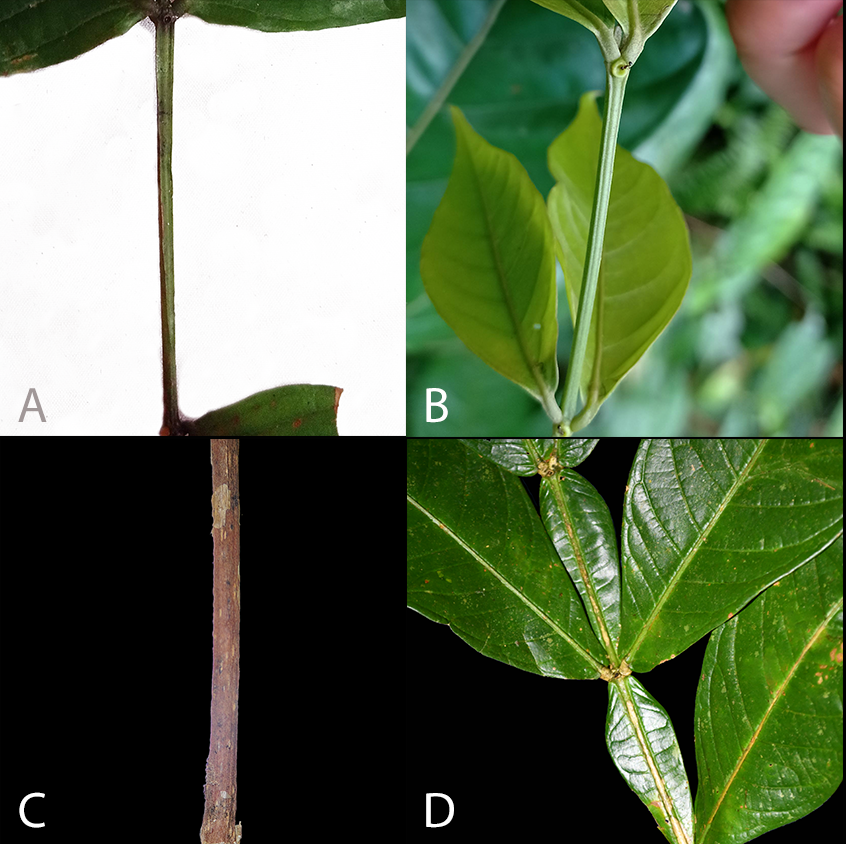


Figure 5. Rachis shapes through *Inga*. A. Channelled; B. Marginate; C. Terete; D. Winged.

- 1. Rachis wing
     1. Wing shape


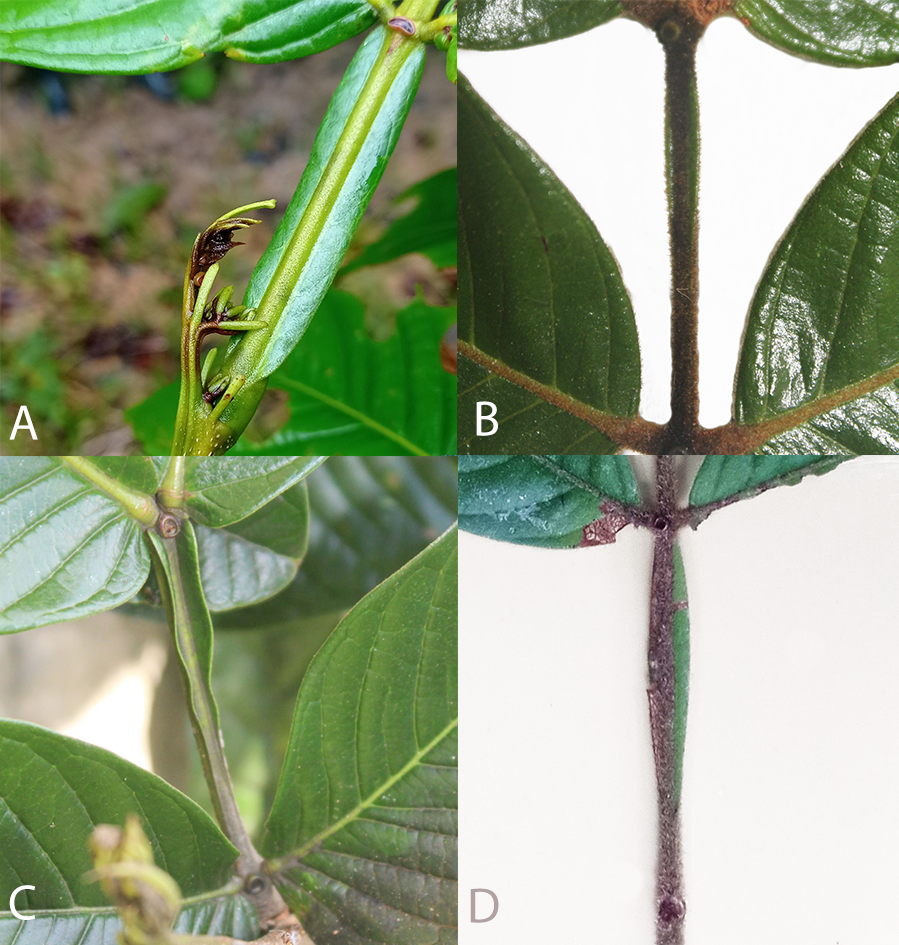


Figure 6. Wing shapes through *Inga*. A. Winged along the entire length of the rachis; B. Winged only below the internode; C. Wings extending from below the internode to mid-rachis; D. Wings extending from below the internode to beyond the mid-rachis.

- 1. Rachis apical appendix
     1. Apical appendix presence


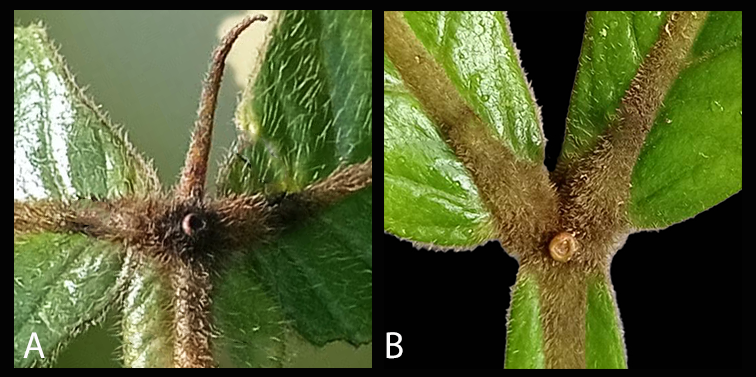


Figure 7. Appendix presence through *Inga*. A. Present; B. Absent.

1. Leaflets
   1. Leaflets shape


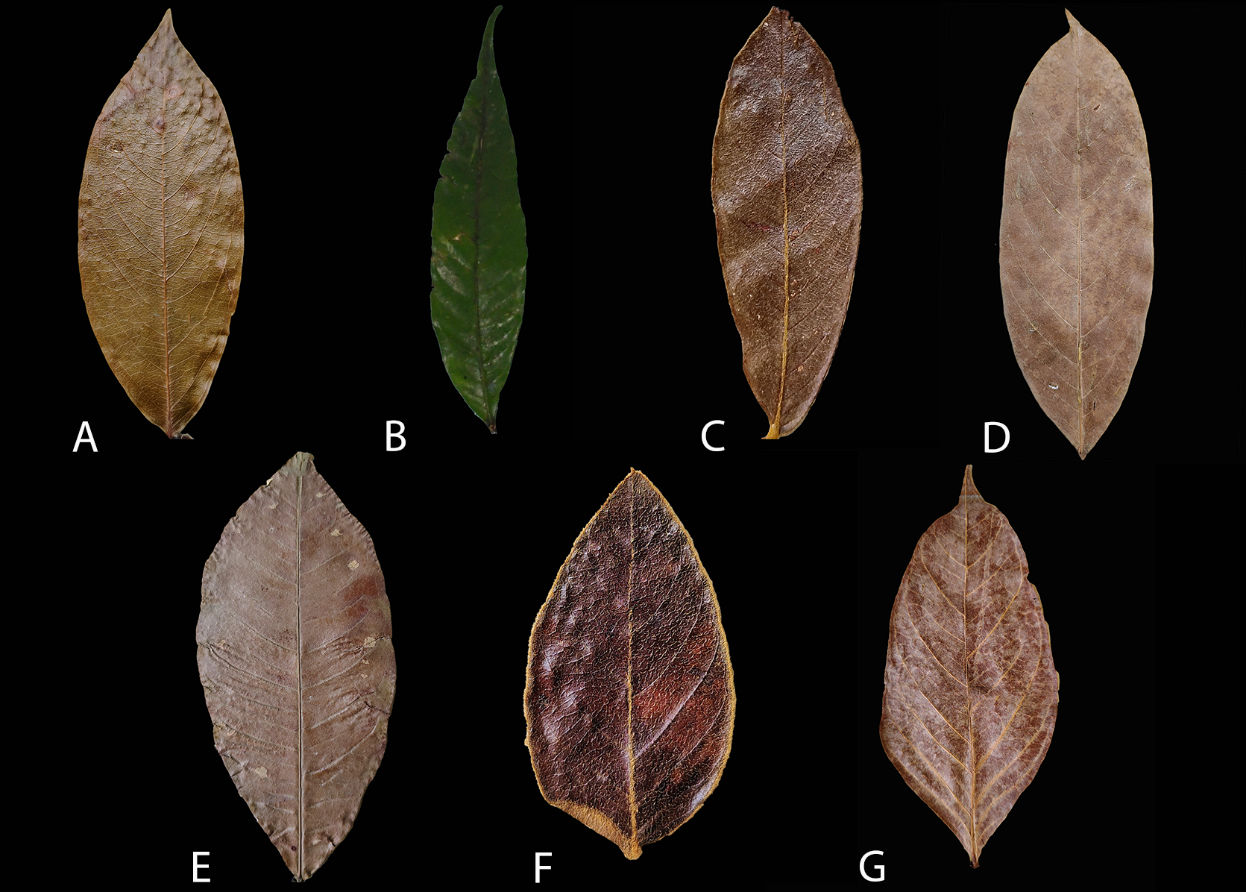


Figure 8. Leaflets shapes through *Inga*. A. Elliptic; B. Lanceolate; C. Oblanceolate; D. Oblong; E. Obovate; F. Ovate; G. Rhomboid.

- 1. Leaflets apex shape


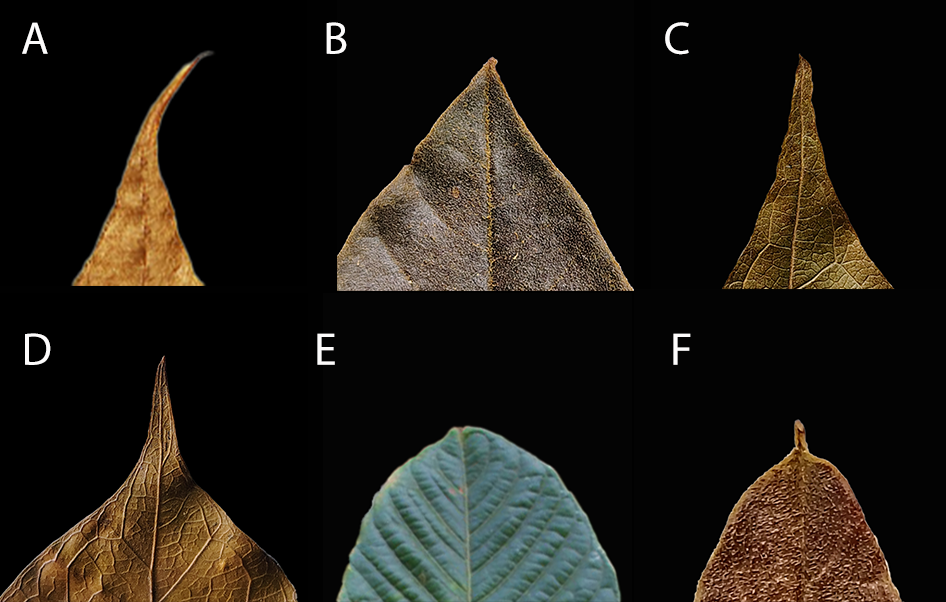


Figure 9. Leaflets shapes through *Inga*. A. Acuminate; B. Acute; C. Attenuate; D. Cuspidate; E. Rounded; F. Mucronate

- 1. Leaflets base shapes


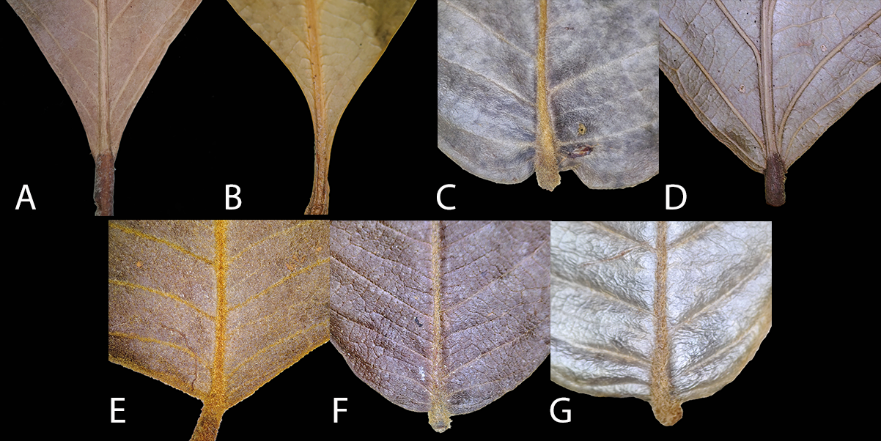


Figure 10. Leaflets base shapes through *Inga*. A. Acute; B. Attenuate; C. Cordate; D. Cuneate; E. Obtuse; F. Rounded; G. Truncate.

1. Indumentum (applies to twigs, petioles, adaxial, and abaxial sides of the leaflets)
   1. Presence of indument


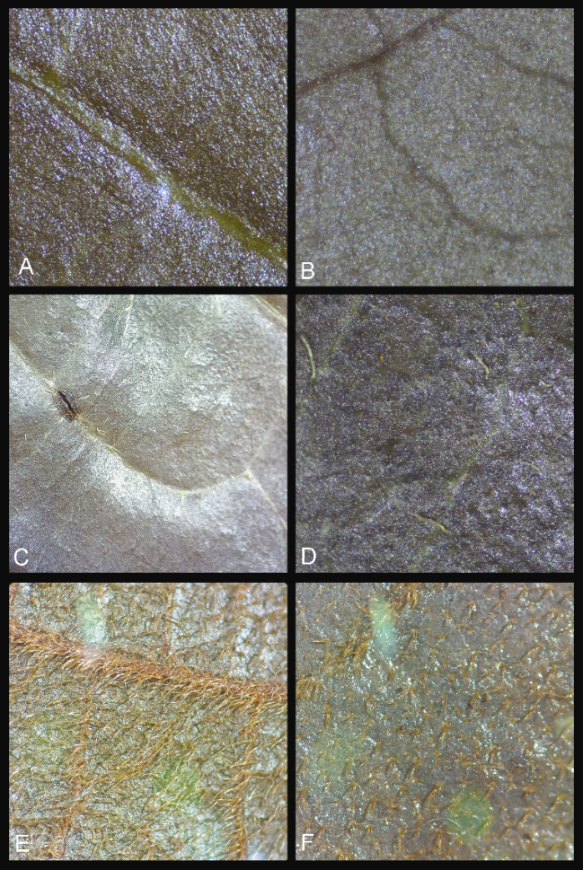


Figure 11. Indumentum presence through *Inga.* A-B. Glabrous; C-D. Subglabrous; E-F. Indument present.

6.2. Type of indument


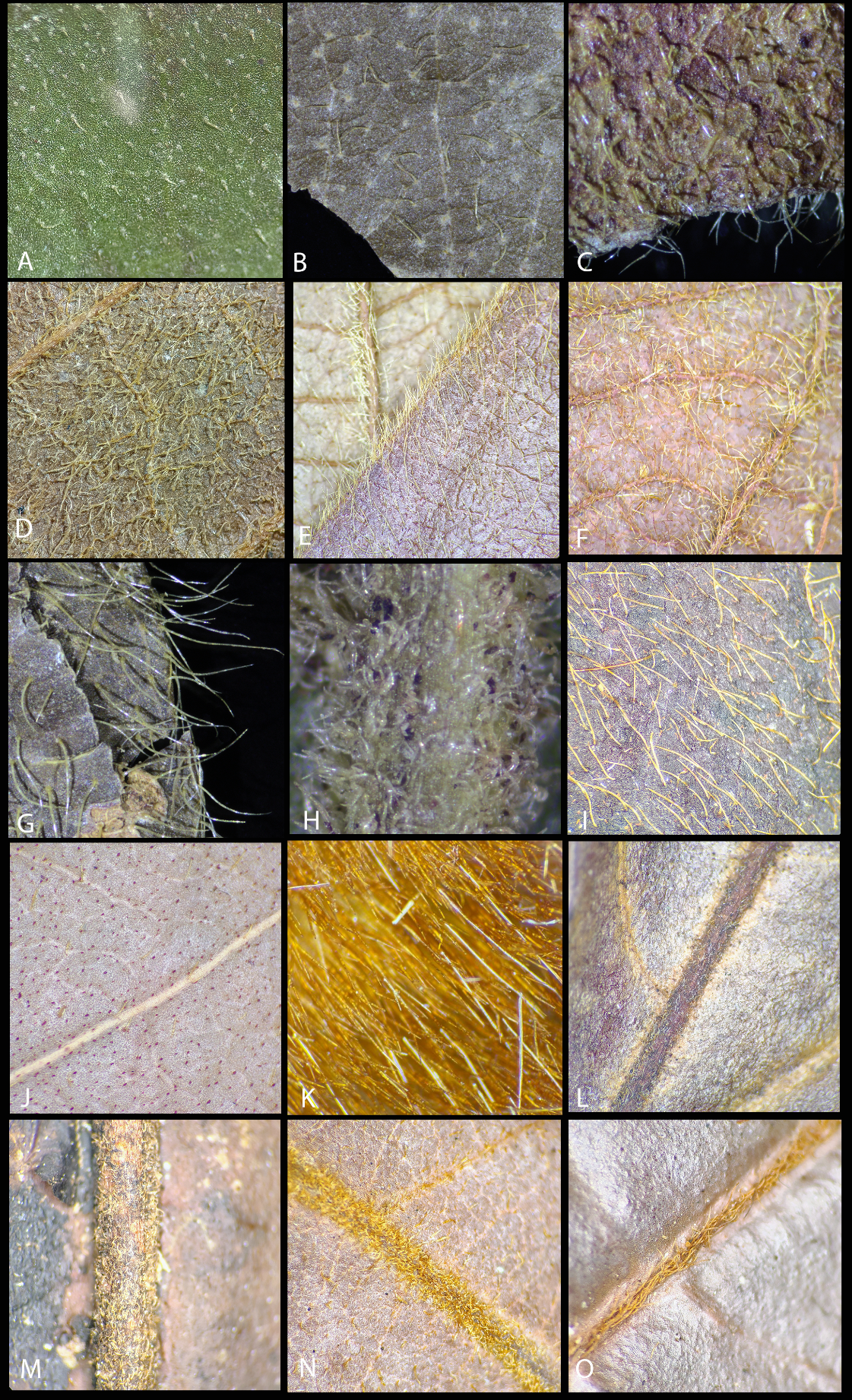


Figure 12. Indumentum type through *Inga.* A. Strigose; B. Tomentose; C. Pubescent; D. Hispid; E. Villose; F. Hirsute; G. Setose; H. Crisped-pubescent; I. Velutinous; J. Minute reddish glandular hairs; K. Stiffy; L. Midrib pubescent; M. Midrib puberulous; N. Midrib tomentose; O. Midrib hispid

1. Nectaries
   1. Interfoliar nectaries attachment


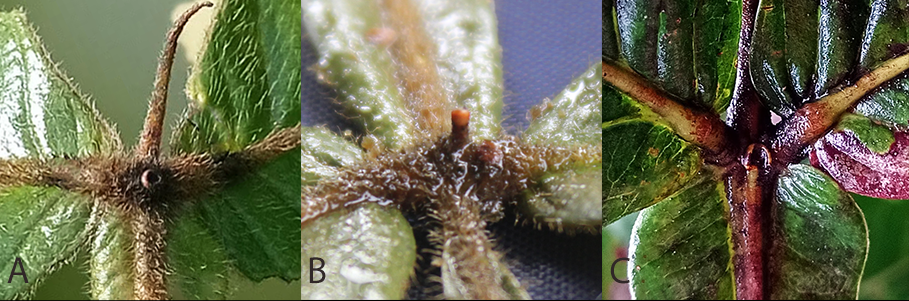


Figure 13. Interfoliar nectaries attachment through *Inga.* A. Sessile; B. Stalked; C. Sunken

- 1. Interfoliar nectaries shape


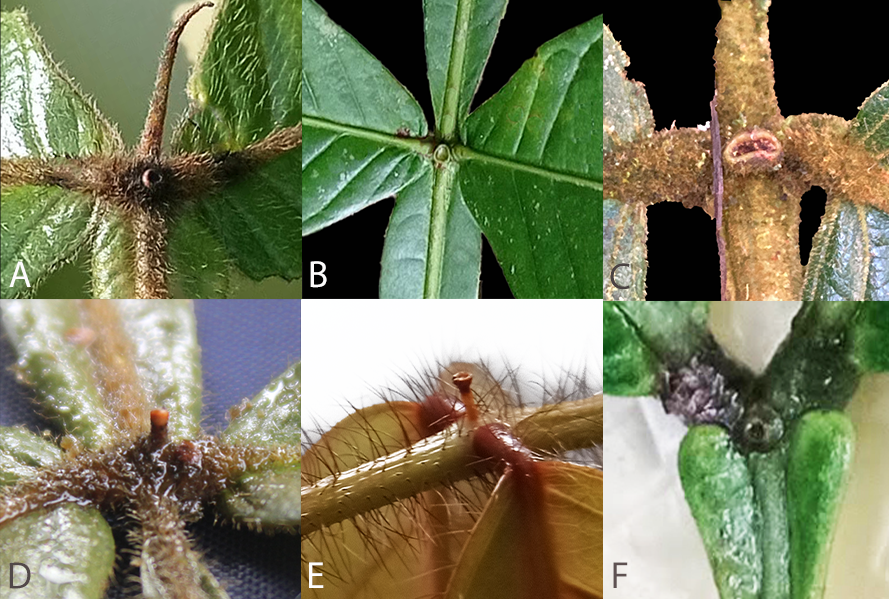


Figure 14. Interfoliar nectaries shape through *Inga.* A. Cup-shaped; B. Patelliform; C. Transversely compressed (kidney shape); D. Tubular with unexpanded head; E. Tubular with expanded head; F. Pulvinate.

- 1. Nectaries special features
     1. Nectaries special features


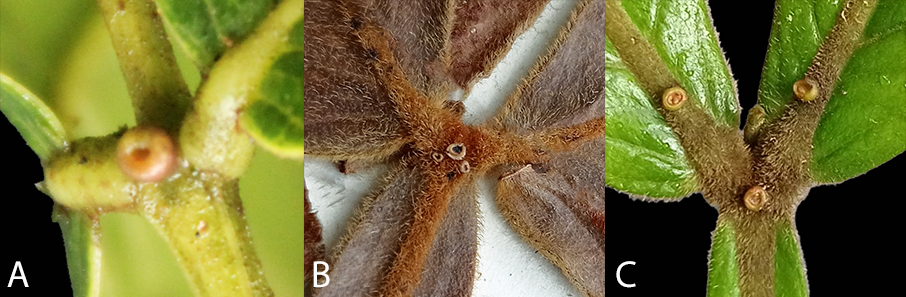


Figure 15. Nectaries special features through *Inga.* A. Thick-walled; B. Multiple nectaries in the internode; C. Additional nectaries on the leaflets.

1. Inflorescence

8.1. Inflorescence position


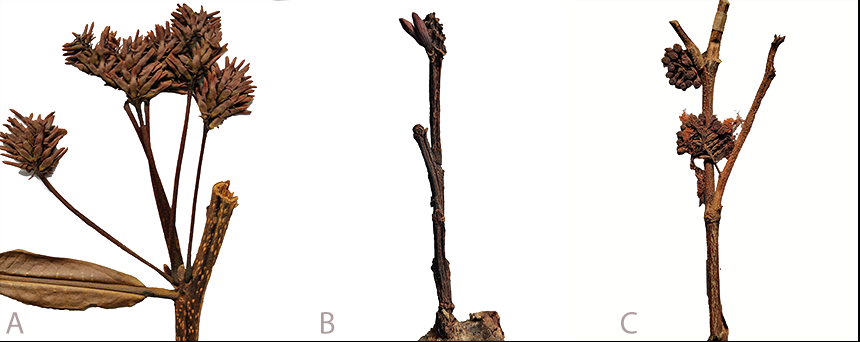


Figure 16. Inflorescence position through *Inga*. A. Axillary; B. Cauliflorous; C. Ramiflorous.

8.2. Inflorescence type


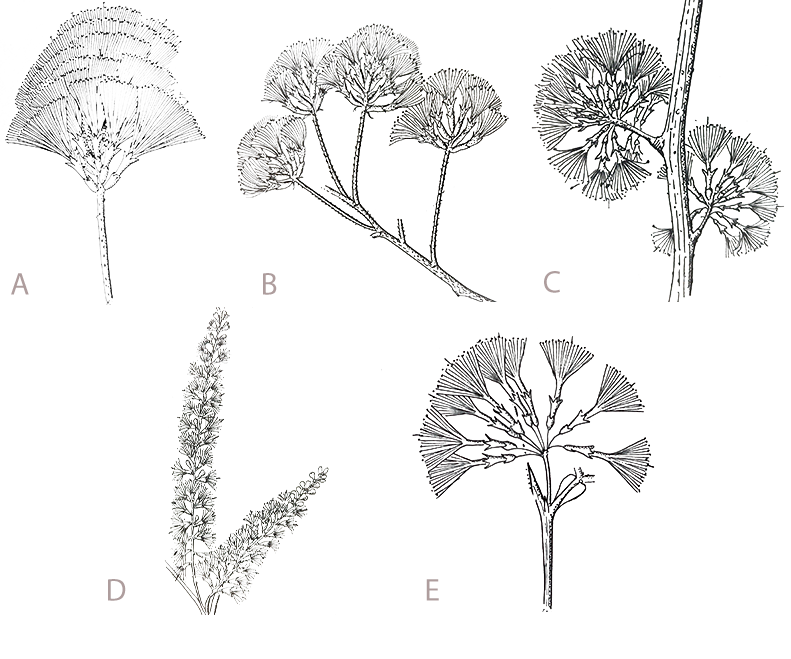


Figure 17. Inflorescence type through *Inga*. A. Capitata; B. Subcapitata; C. Raceme; D. Spike; E. Umbellate. From Pennington 1997.

1. Calyx

9.1 Calyx shape


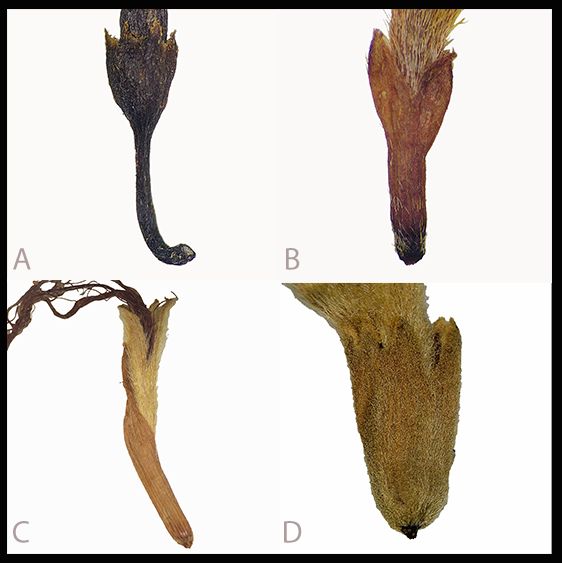


Figure 18. Calyx shape through *Inga*. A. Cup-shaped; B. Funnel-shaped; C. Lateral slit; D. Tubular.

1. Fruit

10.1. Fruit curvature


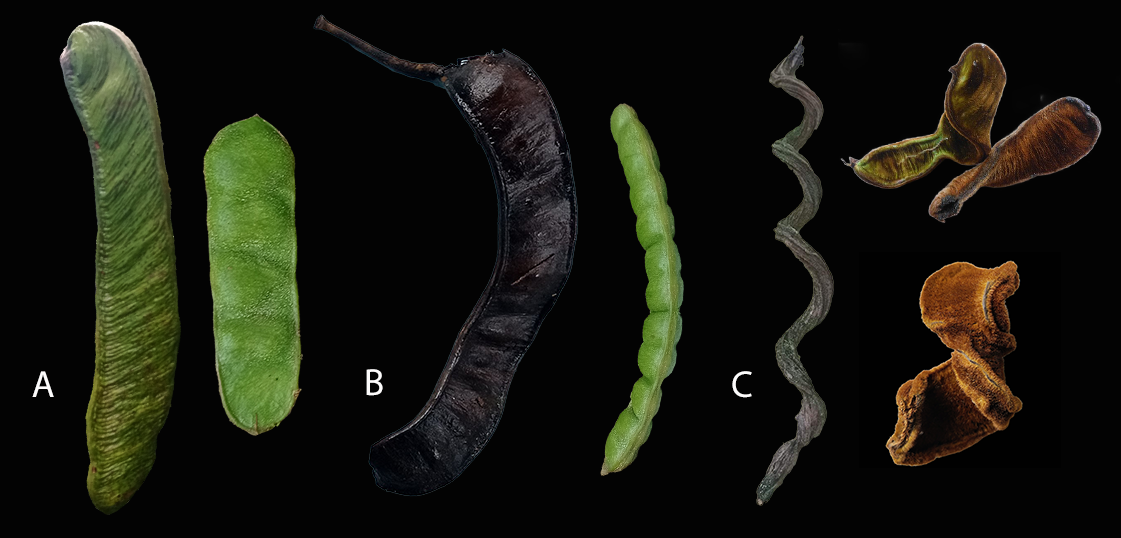


Figure 19. Fruit curvature through *Inga*. A. Straight; B. Convex; C. Spirally/twisted.

10.2. Fruit cross-sectional shape


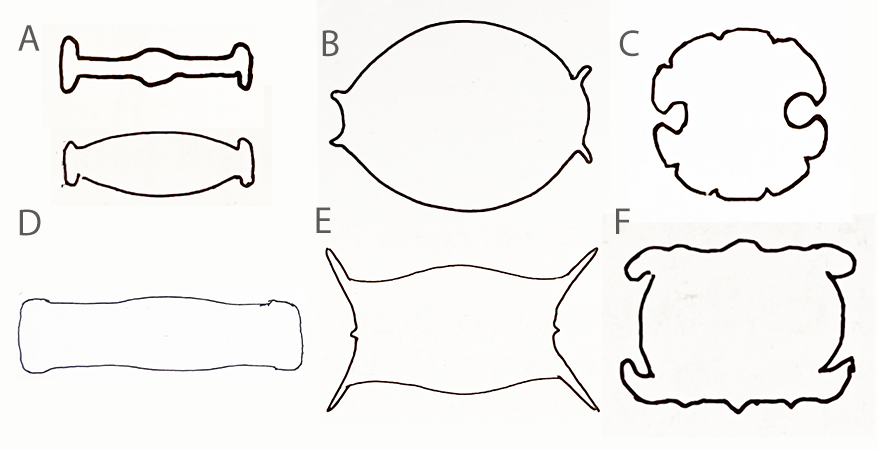


Figure 20. Fruit cross-sectional shape through *Inga*. A. Flat; B. Cylindrical smooth; C. Cylindrical channelled; E. Quadrangular smooth; F. Quadrangular with winged margins; G. Quadrangular channelled.
